# Two distinct actin waves correlated with turns-and-runs of crawling microglia

**DOI:** 10.1371/journal.pone.0220810

**Published:** 2019-08-22

**Authors:** Taeseok Daniel Yang, Kwanjun Park, Jin-Sung Park, Jang-Hoon Lee, Eunpyo Choi, Jonghwan Lee, Wonshik Choi, Youngwoon Choi, Kyoung J. Lee

**Affiliations:** 1 School of Biomedical Engineering, Korea University, Seoul, South Korea; 2 School of Engineering, Brown University, Providence, Rhode Island, United States of America; 3 Department of Bio-Convergence Engineering, Korea University, Seoul, South Korea; 4 Center for Molecular Spectroscopy and Dynamics, Institute for Basic Science (IBS), Seoul, South Korea; 5 School of Mechanical Engineering, Chonnam National University, Gwangju, South Korea; 6 Department of Physics, Korea University, Seoul, South Korea; The University of British Columbia Life Sciences Institute, CANADA

## Abstract

Freely crawling cells are often viewed as randomly moving Brownian particles but they generally exhibit some directional persistence. This property is often related to their zigzag motile behaviors that can be described as a noisy but temporally structured sequence of “runs” and “turns.” However, its underlying biophysical mechanism is largely unexplored. Here, we carefully investigate the collective actin wave dynamics associated with the zigzag-crawling movements of microglia (as primary brain immune cells) employing a number of different quantitative imaging modalities including synthetic aperture microscopy and optical diffraction tomography, as well as conventional fluorescence imaging and scanning electron microscopy. Interestingly, we find that microglia exhibit two distinct types of actin waves working at two quite different time scales and locations, and they seem to serve different purposes. One type of actin waves is fast “peripheral ruffles” arising spontaneously with an oscillating period of about 6 seconds at some portion of the leading edge of crawling microglia, where the vigorously biased peripheral ruffles seem to set the direction of a new turn (in 2-D free space). When the cell turning events are inhibited with a physical confinement (in 1-D track), the peripheral ruffles still exist at the leading edge with no bias but showing phase coherence in the cell crawling direction. The other type is “dorsal actin waves” which also exhibits an oscillatory behavior but with a much longer period of around 2 minutes compared to the fast “peripheral ruffles”. Dorsal actin waves (whether the cell turning events are inhibited or not) initiate in the lamellipodium just behind the leading edge, travelling down toward the core region of the cell and disappear. Such dorsal wave propagations seem to be correlated with migration of the cell. Thus, we may view the dorsal actin waves are connected with the “run” stage of cell body, whereas the fast ruffles at the leading front are involved in the “turn” stage.

## Introduction

Crawling of eukaryotic cells is a complex phenomenon involving many coordinated biochemical events of membrane protrusion, adhesions, detachments and cytoskeletal restructurings. Many significant understandings have been made regarding the biochemical components of cell migration since the pioneering works of [1–4]. The protrusion of leading front results from actin filament polymerization pushing against the cell membrane [5–7]. Then the leading part of a migrating cell form local adhesion sites to the substrate [8–12]. In the rear end, the dissociation of focal adhesions and actin depolymerization, accompanied by cytoskeletal contraction, ensue. Of all these important components for cell crawling, in this paper we are interested in the role of actin polymerization/depolymerization dynamics, in particular, their spatiotemporal dynamic features in association with unusual motile behavior of freely crawling microglial cells.

Previously, a few different types of ‘actin dynamics’ were identified and characterized in connection with cell crawling. For example, Ponti et al. [4] reported that there are at least two different types of actin cytoskeleton kinetics: One for lamellipodium, very narrow zone spatially being confined within 1 ~ 3 microns from the leading edge; and the other for lamella, which is the main cell body. They found that the actin monomer recycling at the leading edge is a lot faster than that of lamella and concluded that persistent crawling depends on the expansion of lamella network, and the faster lamellipodium actin kinetics at the front alone is not sufficient for maintaining directionally persistent movement. On the other hand, in a recent paper [13], Krause and Grautreau stated that lamellipodial persistence is a key factor as for keeping directional persistence.

**Fig 1:**
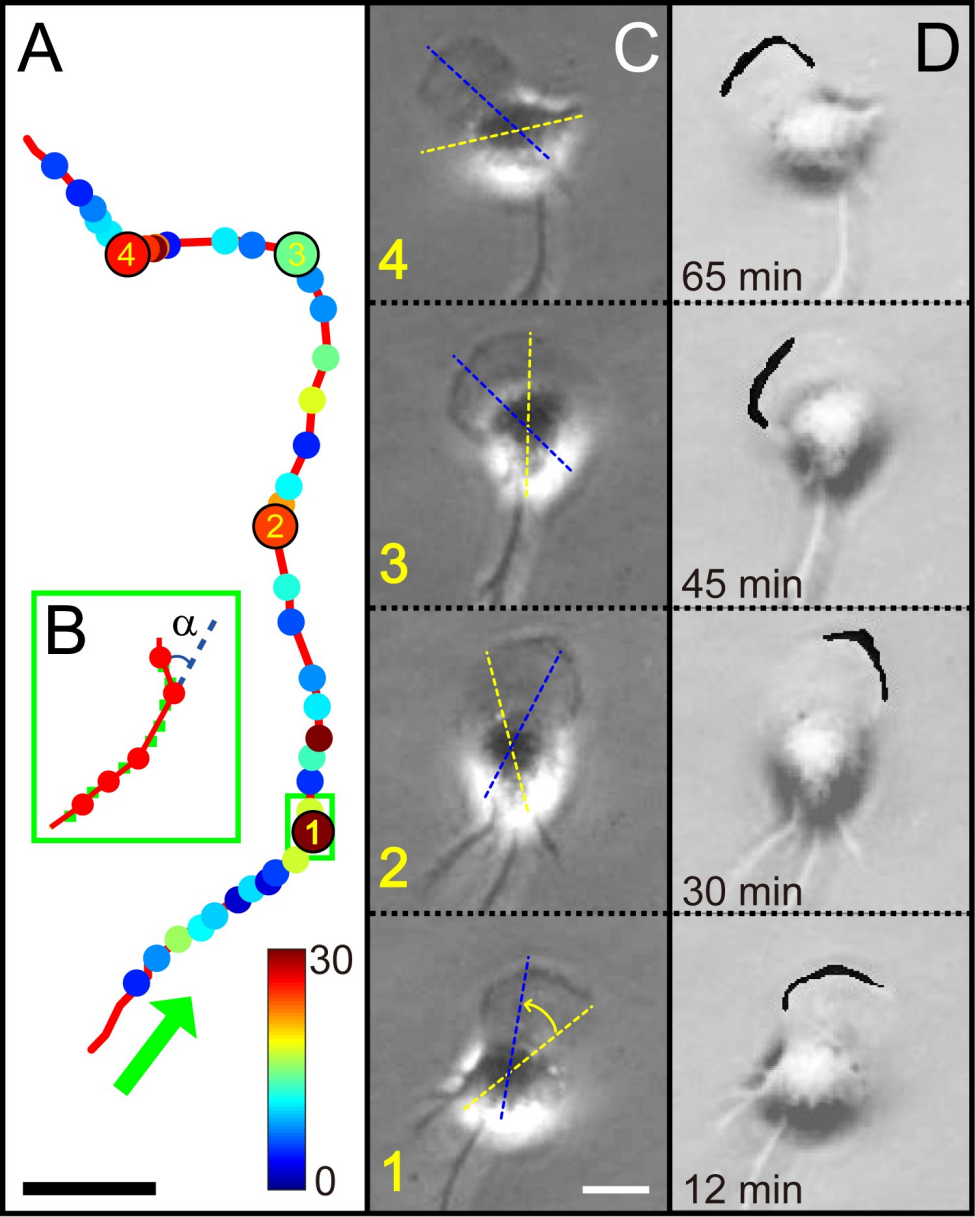
Zigzag turns made by a freely crawling microglia cell and alternating active zone of peripheral ruffles at its leading front. (A) A typical zigzag trajectory is smoothed by the third order polynomial function as a low-pass filtering, which is based on sequential images sampled at an interval of 15 s for 90 minutes (see Visualization 1). Individual turning points are designated by the dots of which colors show the turning angles. Four circles labeled with the numbers (1 to 4) correspond to the time points (12, 30, 45, and 65 minutes in that order). (B) Blown up image showing a sequence of small zigzag turns (green boxed area in (A). The green dots are the centroid positions before the filtering and the red dots mark turning events when a turning event occurs with an angle *α*. (C) Snapshot images showing the typical morphology of a crawling microglia obtained by PCM. Each image corresponds to one of the four labelled positions in (A). Dotted yellow and blue lines superimposed on images delineate the previous and new directions, respectively. (D) Images highlighting the active zone of peripheral ruffles along the leading front. For the sake of clarity, the active zone of peripheral ruffles is indicated with a saturated black (through thresholding and binarizing) and it is superimposed on (C) images. Scale bars in (A) and (C) are 10 and 20 μm, respectively.

**Fig 2:**
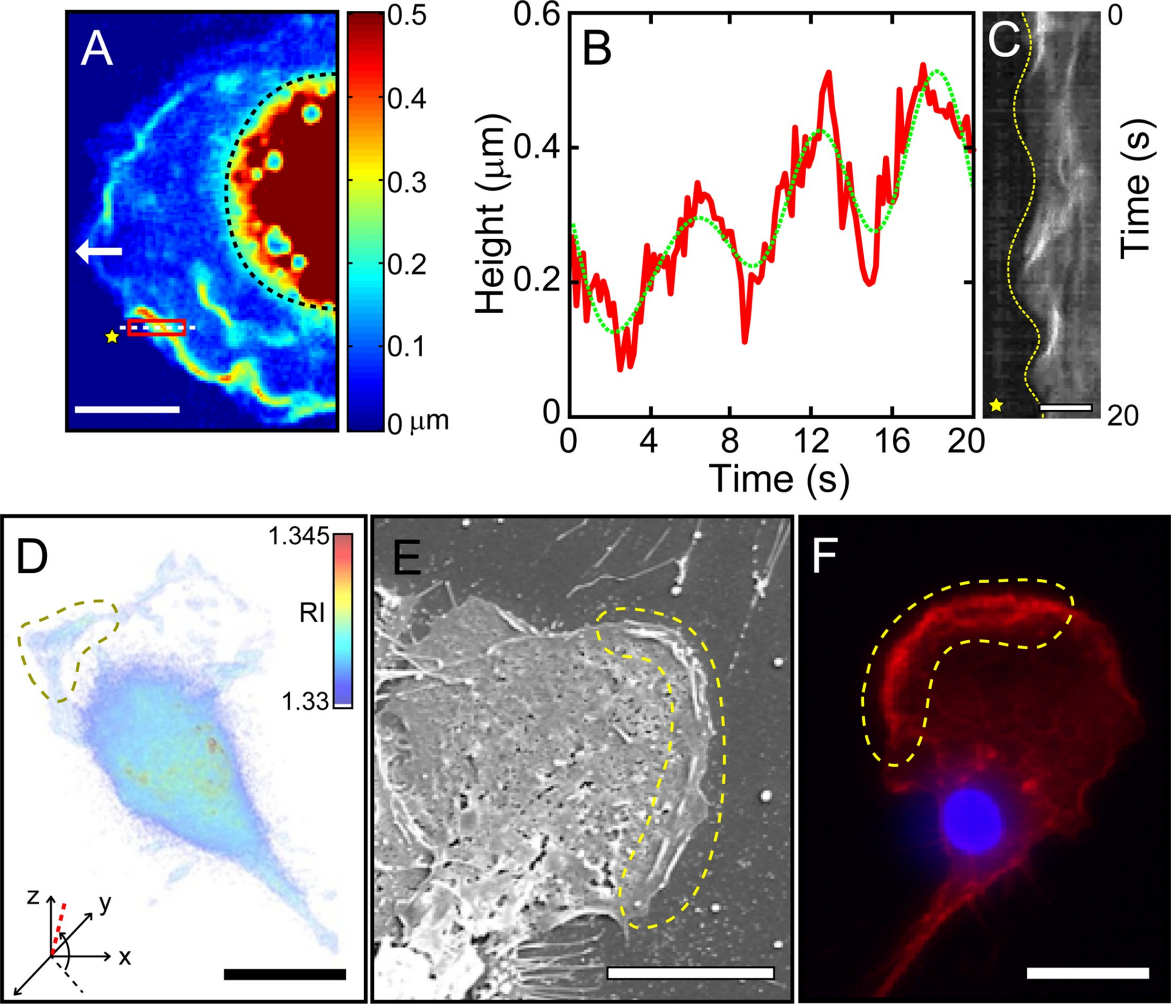
Peripheral ruffles waves of crawling microglia cells visualized by three different imaging modalities. (A) SAM phase image of peripheral ruffles in living microglia obtained by a QPI technique. The processed image is a heat map of the height of the dorsal surface of a MG cell from the substrate (white arrow: moving direction; black dotted line marks the core area; see Visualization 2 for their spatiotemporal evolution). (B) Averaged height (of the red-boxed area in (A)) vs. time, showing periodic nature of ruffle waves (the green dotted line is a boxcar-filtered (over 10 points) trace. (C) Kymograph showing spatiotemporal evolution of a leading front alongside the oscillatory behavior of ruffle waves (the 1D space of the kymograph is taken along the white dotted line in (A); the yellow dotted line traces the position of the leading front; and the yellow star marks the space outside the cell). (D) Isosurface image of 3-D RI tomogram (see Visualization 3). The bounded area by yellow dotted line is where peripheral ruffle wave dynamics are active (the tomogram viewing direction is indicated by a red dotted line in the x-y-z coordinate system with the viewing angles, -137° (azimuth) and 84° (elevation)). (E) SEM image of a fixed microglia showing the existence of ruffle waves at the leading edge (in the region bounded by yellow dotted line). (F) Epi-fluorescence image of a fluorescence stained (fixed) microglia showing F-actin (red) and core nucleus (blue, DAPI). Scale bars in A), C), D), E) and F) are 10, 5, 20, 10, 20 μm, respectively.

The two different actin kinetics working at two different time scales of a cell crawling often support complex spatiotemporal wave activities. The fast, small scale, actin waves at the very leading edge are generally coined as membrane ruffles: They spontaneously emanate from the leading edge, travel around the cell periphery, sometimes, fold back onto itself and crease [14, 15]. It is known that the balance of F-actin branching versus elongation controls lamellipodial persistence and protrusion speed [13]. The actin dynamics within the lamella (i.e., cell body) can also support complex actin waves. Often, waves of actin polymerization/depolymerization, which are physically much larger than the ruffles at leading edge, start out near the leading edge and travel centripetally toward the core region of the cell body, either on the dorsal or ventral membrane. Earlier experiments have identified some key factors [3, 13, 16], such as the Rho family of GTPage and the Par complex, contributing to the formation of leading edge and maintaining the front-rear axis of a migrating cell. However, the coordination between the aforementioned two different actin kinetics and the relationship between two different types of actin wave dynamics remain as on-going problems to be answered. Moreover, how the spatiotemporal actin wave dynamics translates into a particular motile path of a freely crawling cell body is far from being clear. A few years ago, we made an interesting experimental observation regarding the motile behavior of microglia (MG) cells [5]. In the absence of any external stimulus, the trajectories of freely crawling cells on a flat substrate could be viewed as a noisy, yet temporally correlated, sequence of small run-and-turns (see Figs 1A and 2B). The average inter-turn time interval was around 2 minutes. Cells seemed to have a built-in mechanism for a short-term memory on the direction of previous turns it made, thus setting their zigzag motility [5]. It turns out that this behavior is quite general for many different crawling cells, including Dictyostelium discodium amoebae, fibroblasts, and keratocyte cells [6, 7, 17], although the duration of memory time and regularity in the distribution of inter-turn intervals strongly depend on the cell type.

In this study we employ a highly accurate and sensitive quantitative phase imaging based on a synthetic aperture method (SAM) [18, 19] along with an optical diffraction tomography (ODT) reconstruction [20, 21] to visualize 3-D actin waves and monitor their spatiotemporal evolutions in connection with the motile behavior of a crawling MG cell. Importantly, we could identify two different types of actin waves, both of which are noisy but temporally periodic. One is peripheral ruffle waves having a short (~ 6 s) period. They are spontaneously excited vigorously at some portion of cell boundary to guide the cell into a particular direction, thereby may initiate a turn. The other is dorsal actin waves propagating from the front area of lamellipodium to the cell nucleus in a periodic manner of approximately 2 minutes. According to our analysis, dorsal actin waves can propagate at a speed of ~ 2.5 μm/min toward the core of the cell while the cell migrates in the opposite direction with a similar speed. In fact, the velocity of wave propagation and that of cell migration seems to be correlated. In short, “runs” are connected with slow dorsal actin waves whereas “turns” seem to be initiated by ruffle waves at the leading edge. In other words, the emergence of ruffle waves could be viewed as a precursor of the new direction to which the cell moves. Moreover, when the cell crawling is physically confined within a narrow linear track (with groove), its peripheral ruffles at the leading front become spatially synchronized; they do not travel back and forth along the leading front. Within the confined linear track, however, the directed cell migration is not hindered at all.

## Materials and methods

### Primary cell culture of microglia

All the animal experiments in this study were approved and confirmed by our institutional review board for animal research, the Korea University Institutional Animal Care and Use Committee (KUIACUC-20170405-2) and all experiments were performed in accordance with relevant guidelines and regulations. Detail description of pure microglial cell culture was given in ref. 1. Briefly, we prepared primary glia co-cultures from the cerebral cortex of postnatal day 1 ~ 2 Sprague Dawley rat (Charles River, OrientBio Inc.) which is euthanized by decapitation. After cultivating them in a T-75 culture flask (37°C, 5% CO_2_) containing DMEM medium supplemented with 10% fetal bovine serum and 1% penicillin streptomycin at an initial seeding density of about 2.5 × 106 cells/flask for two weeks, cells in a T-75 culture flask were shaken at 280 rpm for 20 minutes to harvest microglial cells detached from the surface of glial cells fully grown on a flask substrate. The supernatant was collected and then centrifuged at 1,500 rpm for 5 minutes to obtain microglial cells deposited at the bottom of the tube. Finally, microglial cells were plated onto poly-D-lysine coated coverslip at density of 50 cells/mm^2^ for experiments.

### Quantitative phase image with synthetic aperture method and optical diffraction tomography

Biochemical compositions of actin waves are already well characterized with advancements in various fluorescent molecular markers and related imaging tools [15, 22, 23]. However, like any other fluorescent imaging experiments fluorescence-based imaging techniques can suffer from phototoxicity and photobleaching which can interfere with normal cell functions including the actin dynamics. However, the advantage of label-free quantitative phase optics is two-fold: first of all, it is non-invasive to the cell, thus, ideal for a long-term time-lapses imaging with no worry on photo-toxicity or bleaching; and, second of all, it can produce 3D phase tomography. Of course, fluorescent imaging techniques are useful in seeing various relevant biomolecules (genes and proteins) in actions. In contrast, quantitative phase imaging is good for visualizing delicate 3D structures of phase objects like actin waves that are associated with crawling cells. For those reasons, often it is not suitable for long-term (> 1 day) live-cell imaging. Similar issues also exist with the imaging modality such as scanning electron microscopes (SEMs) and atomic force microscopes, with which one can obtain quantitative 3-D images, but only when cells are treated and no longer alive [24, 25]. On the other hand, quantitative phase imaging (QPI) methods have been recently proven to be useful for quantifying physical properties (structures) of translucent samples such as individual living cells or tissues without any exogenous labeling agents. Typically, transmitted phases are measured over specimens and processed afterwards to reveal sub-cellular structures. QPI methods are not only fast enough for measuring dynamic structural changes taking place inside cells, for example, high-frequency membrane fluctuation of red blood cells [26–28] and transmembrane water fluxes in neurons [29, 30], but also sensitive and accurate enough to measure the dry mass of chromosomes [31, 32]. Furthermore, if combined with scanning systems, QPI methods can map out 3-D images of refractive index of living cells to identify its internal structures with a high spatial resolution and sensitivity. In fact, they could be used as an optical biopsy tool for identifying cancer cells inside densely-packed tissues and calcifications in the breast [33]. With these capabilities, QPI can potentially be very useful for quantifying subcellular actin waves in living cells and thereby facilitate a better understanding of their roles in physiological and pathological function of cells.

In this work SAM was used to obtain 3-D shape information of ruffle waves supported by a crawling cell [19]. An interference microscope with a sample and a reference arm was built using a coherent light source (He-Ne laser, λ = 632 nm). Variation of the interferogram introduced by a living cell in the sample plane was then recorded in the detection arm designed as an off-axis configuration. An oil immersion objective lens (Olympus, 100X, 1.4 NA) was used to capture the light transmitted through the sample. A total of 40 images were taken while a 2-D scanning mirror placed at the front conjugate plane of the sample steered the illumination angle ranging from 0 to 0.6 NA for 200 ms. All images were processed into electric field images according to the digital holographic method and superposed in a coherent fashion to produce final synthesized images. In addition, the diffraction noise on top of bare coherent images was considerably suppressed by the synthetic process. To reconstruct 3-D image with ODT, a total of 200 images were obtained with various illumination angles using the 2-D scanning mirror from 0 to 1.4 NA for 1.33 s. Using the reconstruction algorithm based on the Fourier diffraction theorem under the Rytov approximation and the non-negative constraint iteration, 3-D image was then retrieved from the measured optical field involved in refractive index information [18–21].

### Fluorescence cell images

For fixation, microglia cultured on a cover glass were incubated in 4% paraformaldehyde at room temperature for 20 minutes and then treated with 0.1% Triton X-100 (Sigma-Aldrich) for 20 minutes at room temperature. After rinsing with phosphate-buffered saline (PBS), cells were incubated with rhodamine-phalloidin (Sigma-Aldrich) at 1:200 dilution and DAPI (4’,6-diamidino-2-phenylindole, Sigma-Aldrich) at a concentration of 0.1 μg/mL in PBS for 20 minutes to stain cytoskeletal F-actin and cell body, respectively. Fluorescence cell images were taken with a confocal microscope (Axio Observer D1; Carl Zeiss, Germany).

### Scanning electron microscopy

Microglia were fixed in Karnovsky's fixative (2% glutaraldehyde and 2% paraformaldehyde in 0.05 M sodium cacodylate buffer; pH 7.2) for 2 hours at 4°C. These cells were then dehydrated in stepwise increase of ethanol concentrations from 30% to 100% at intervals of 10%. Subsequently, samples were dried at 60°C for 24 hours and finally coated with platinum (Pt) using a sputter (Sputter E-1030, Hitachi, Japan). Scanning electron microscopy imaging (SEM; FE-SEM S-4700, Hitachi, Japan) was performed at 15 k.

### Time-lapse imaging with a phase-contrast microscopy

Substrates containing harvested MG were placed at 37°C with 5% CO_2_ regulated in a home-built chamber mounted on an inverted microscope (IX71, Olympus, Japan) stage. Time-lapse images were acquired at an interval of 15 seconds, typically for a time period longer than 24 hours, using a cooled CCD camera (MFcool, ProGres, Germany). Phase contrast microscope (PCM) time-lapse images were acquired inside a temperature (37°C, 5% CO_2_) regulated Live Cell Instrument (LCI) customized chamber mounted onto a microscope stage [5].

### Micro-patterned substrate

Micro-scale grooves were patterned with photolithography using a positive photoresist (AZ7200, AZ Electronic Materials Ltd.). These patterned micro-scale grooves (20 μm in width) were etched by deep reactive ion etching (DRIE) and the photoresist layer was removed. Our target depth of DRIE was 4 μm. The fabricated wafer was used as a master for PDMS (Sylgard 184 Silicone Elastomer, Dow Corning) replica molding. After that, PDMS 10:1 mixture was poured over the fabricated wafer and salinized using vapor-phase (tridecauoro-1,1,2,2, -tetrahydrooctyl)-1-trichlorosilane (Sigma Chemical Co., St. Louis, MO, USA) under vacuum for 15 minutes before PDMS pouring. After peeling PDMS from the wafer, we rinsed PDMS with acetone, isopropyl alcohol (IPA), and deionized water (DI water) in order.

### Image analysis of a cell migration

The detailed descriptions of cell tracing and image analysis were given in ref. 5. To trace out the crawling cell in detail, all acquired images were binarized and then the centroid position of cell body was extracted for each frame. Based on the position data of extracted centroids, first the trajectory was smoothed by the third order polynomial function (as a low-pass filtering) in order to minimize some high-frequency fluctuation noise and image processing errors. The polynomial function (third order) was applied to fit the sliding window of 11 successive points (which corresponds to 150 seconds) locally. Next, the local curvature *k* was calculated by curvature function at each time step using the filtered trajectory. Finally, a low-pass filter was applied to *k(t)* with cutoff at 30 seconds, and the local extrema of the filtered *k(t)* were considered to be a turning point, along each segment the turning angle is defined as *α*. Cell images were cropped with the typical cell size area and aligned with respect to the centroid positions and turning angle of cell.

Kymographs were generated from these aligned cell images using ImageJ. For defining ruffling regions at the leading edge, the images were binarized with the threshold grayscale value between 70 and 80 (out of 255).

## Results

### The emergence of biased peripheral ruffle waves leads a turn in crawling MG cells

When microglia are harvested from rat brains and plated on to a culture dish at a very low cell density, they are free from any interactions. Thus, their motilities are solely determined by individual cells themselves. As aforementioned in the introduction, these free individual MG cells exhibit quite an interesting motile behavior as illustrated in Fig 1A. Using low-pass filtering, the trajectory is represented as red line fitted locally from the centroid position data to a third order polynomial function with sliding window of 11 successive points. Next, in this filtered trajectory, the local curvature *k* is calculated by the curvature function at each time step. Finally, *k(t)* is applied with the cutoff at 30 seconds, and the local extrema of the *k(t)* are set as turning points shown as dots in Fig 1A. In each dot, the turning event occurs with an angle *α* and its magnitude is represented by color. In Fig 1B, green dots are the centroid position data (before applying the low-pass filtering) and red dots represent the turning events of the green box shown in Fig 1A. The turning events occur about 50 times for 90 minutes. Typically, a trajectory of the cell can be viewed as a sequence of “runs” and “turns” inter-turn-intervals on about 2 minutes (the average of 1.82 ± 0.69 min) similar to the value of previously published data (1.9 ± 0.2 min). And the segmented red lines (in Fig 1B), composed of two neighboring red dots, have the average angle of 17.29 ± 23.25 degrees. This value is smaller than the previously published data (48.3 ± 7.3 degrees) due to the shorter imaging acquisition time (~ 90 minutes) than before (~ longer than 24 hours).

Additionally, to clearly show the relationship between the turning direction of cell and the active zone of peripheral ruffles at the leading edge, four turning positions which have large turning angle are chosen with the corresponding time points of 12, 30, 45, and 65 minutes in Fig 1A. Their matching PCM snapshot images showing cell turning are given in Fig 1C. We have marked the previous and new directions as yellow and blue dotted lines superimposed on these images, respectively. To make more clear contrast on the active zone, the peripheral ruffles have been delineated with a saturated black color (through thresholding and binarizing) in Fig 1D and superimposed on Fig 1C. When peripheral ruffles show a bias (activated intensively in one side) as shown in Fig 1D, the cell turning follows immediately to the same direction. Hence, we could notice a close relation between the turning direction (blue dotted line in Fig 1C) and the active zone of peripheral ruffles in Fig 1D.

### Periodic oscillatory behaviors of peripheral ruffle waves at the leading edge

Using QPI technique in conjunction with SAM (see Materials and methods for further details), we analyzed the 3-D morphology of peripheral ruffles and their dynamics in a time-lapse mode. Fig 2A is an example showing a color map of crawling microglia where the height (from the substrate to the top of the ruffle) is calculated based on the measured quantitative phase delay information acquired by QPI. Clearly, the cell carries long narrow strands (but in irregular shape) of ruffle waves along the leading front of lamellipodium. To obtain the height, width and length of peripheral ruffles originally segmented in 3-D, the cell image is first projected on 2-D with threshold and binarized. Then, peripheral ruffles are assumed to be segmented in rectangular shapes. From these images, the measured height, width and length of peripheral ruffles are 0.40 ± 0.13 μm, 0.39 ± 0.09 μm and 9.04 ± 2.51 μm (based on 5 cases), respectively. The height of the core area (demarcated by black dotted line) is far beyond the dynamic range of our QPI height measurement of ruffle waves, thus unable to be measured.

These ruffle waves are periodically generated and collapsed by folding back onto the cell body with a period of about 6 seconds as illustrated in Fig 2B (see Visualization 2). In addition to the temporal height modulation, the overall height is gradually increased from 0.1 to 0.5 μm within four consecutive waves. This slight increment is simply due to the fact that the cell has advanced a little (along the direction marked by a white arrow) during our observation.

The oscillatory behavior of peripheral ruffles is also apparent in the kymograph of Fig 2C taken along the center horizontal (white dotted) line inside the red-boxed area in Fig 2A. The lamellipodial boundary oscillation (guided by the yellow dotted line shown in the kymograph of Fig 2C) is in a synchrony with the ruffle height oscillation (green dotted line shown in Fig 2B). The peaks (in both Fig 2B and 2C) show up at 6, 12, and 18 seconds, approximately. The height at peaks and the average oscillating period of peripheral ruffles are 0.40 ± 0.13 μm and 6.45 ± 0.44 seconds, respectively (based on 6 different sampled area along the leading edges of 4 different cells).

We also obtain 3-D refractive index (RI) tomogram as shown in Fig 2D (see Visualization 3) using a standard tomographic algorithm [18, 20]. The tomogram, the isosurface image of RI, also well illustrates that peripheral ruffles (the region bounded by yellow dotted line in Fig 2D) are active not over the entire front area, but only in part of it. Tomogram viewing angles are -137° and 84°, azimuth and elevation, respectively. In addition, the features of peripheral ruffle waves visualized by our QPI technique are compared with those obtained with other imaging modalities such as SEM and fluorescence microscope. The SEM image of Fig 2E clearly shows surface protrusion of these peripheral ruffles (see the area bounded by yellow dotted line) as well as some internal cytoskeletal network structures. The fluorescent image of Fig 2F further confirms similar ruffle structures that are rich in F-actin fibers (see the area bounded by yellow dotted line). Taken all images and movies acquired through four different imaging modalities together, we could conclude that peripheral ruffle waves are closely associated with actin polymerization/depolymerization taking place at the moving front. They only form in the leading edge of the front and oscillate with an approximate period of 6 seconds.

### Periodic dorsal waves analogous to forward migrating features in crawling cells

**Fig 3:**
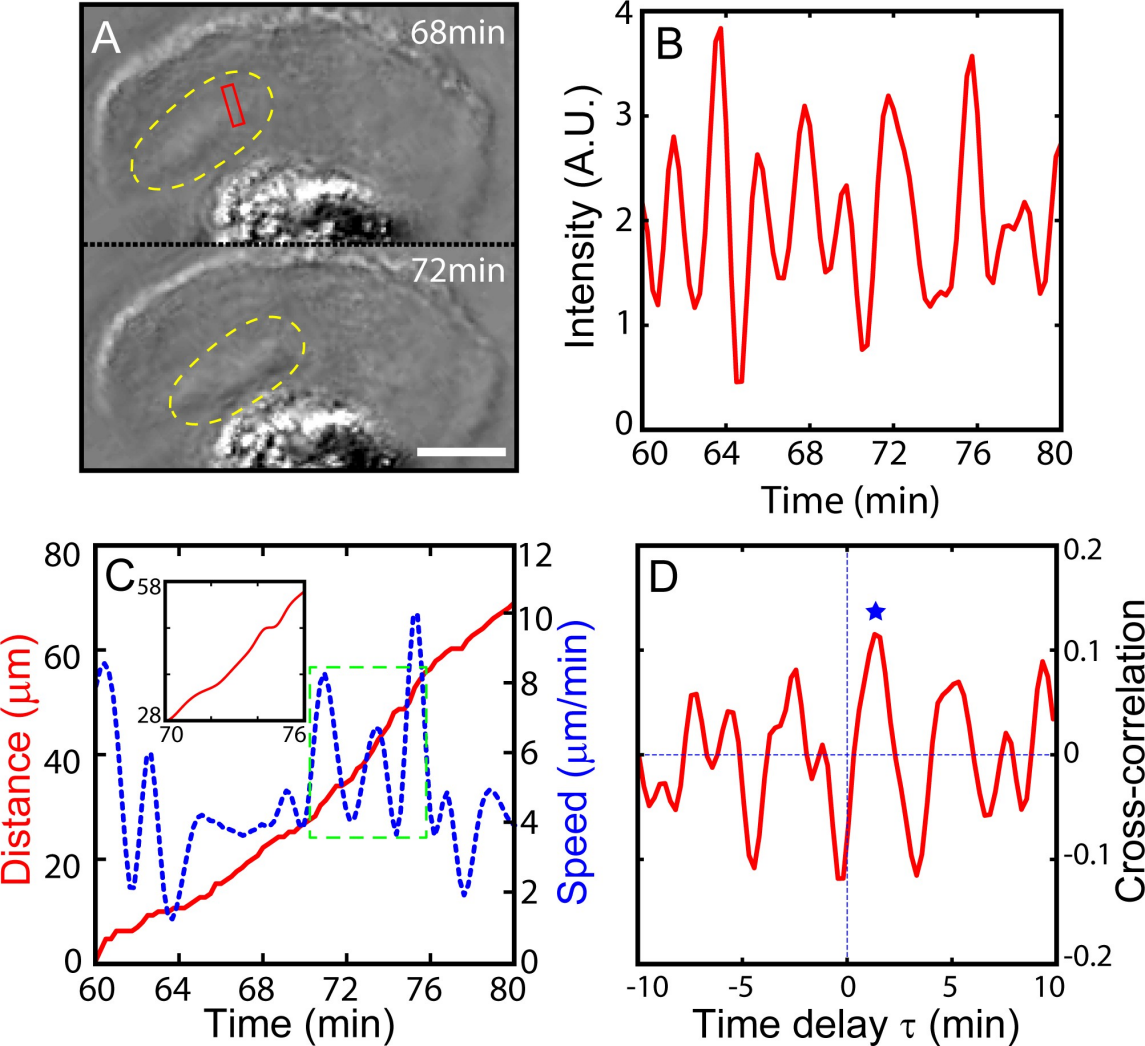
Correlation between periodic dorsal actin waves and the pulsatile movement of crawling MG cell body. A) PCM snapshot images showing a dorsal actin wave t = 68 and t = 72 minutes (dorsal actin wave is marked by yellow dotted line, see Visualization 4). B) The oscillatory average intensity (of the pixels inside the red box) time series indicating the periodic passage of dorsal actin waves travelling from the front to the cell body. C) Plots of the distance (red line) travelled by the crawling MG and its speed (blue dotted line). The inset is simply a close-up view of the distance inside the box bounded by the green dotted line. D) Cross-correlation function *Cτ* of the intensity fluctuation of dorsal actin wave and moving speed of crawling cell body. The blue star marks the first positive peak. Scale bar of A) is 10 μm. Time series in B) and C) are based on a sequence of time-lapse images acquired at a time interval of 15 s.

Our QPI data also show that, from the lamellipodium just behind the leading front, where peripheral ruffle waves exist, the travelling bands that are high in F-actin emanate once in a while and propagate toward the core of the crawling cell. We name them “dorsal waves”. Two snapshot images acquired through a PCM are shown in Fig 3A, depicting such a wave while the cell is moving (see Visualization 4). Dorsal waves are generated repeatedly while the cell body is in motion. In fact, the time series in Fig 3B well illustrates a periodic nature of the dorsal wave propagation with a period of about 2 minutes (1.91 ± 0.87 minutes). Dorsal wave ‘packets’ vary in their shapes as they are created at the leading edge and travel from different directions toward the cell centroid. Therefore, the time series of average intensity (in Fig 3B) acquired at the marked location (red bounded box in Fig 3A) fluctuates unevenly. On the other hand, by tracing the centroid position of the crawling cell (see in Ref. 5), the travelling distance is acquired, and then each frame of cell image is aligned. A typical example is shown in Fig 3C. Interestingly, the microglia move in a pulsatile fashion, alternating between a fast-run and a slow-run approximately every 2 minutes (as a period of 1.82 ± 0.96 minutes). The cell body moving speed vary and oscillate periodically with the fast-run at 10 μm/min and the slow-run at 2 μm/min. These pulsatile movements (fast-run and slow-run) of cell appears to be in a time span similar to the period of dorsal waves. Subsequently, to quantify the relationship of these two oscillatory phenomena, we calculate the cross-correlation function as the following equation:

$$C_{\tau }=\frac{1}{N}\frac{\sum_{i}(x_{i}-\langle x\rangle )(y_{i+\tau }-\langle y\rangle )}{\sigma_{x}\sigma_{y}}.$$ (1)

where N is the total number of samples and *τ* is the time delay between the two signals, the speed of cell body *x*_*i*_ and the intensity of observed ruffle waves at a single position *y*_*i*_, 〈*x*〉, (or 〈*y*〉) and *σ*_*x*_ (or *σ*_*y*_) are the mean and standard deviation of *x*_*i*_ (or *y*_*i*_). As shown in Fig 3D, the correlation function has a primary peak (marked by a blue star) at around *τ* = 1.4 minutes, meaning the dorsal wave propagation (at the red-boxed area in Fig 3A) advances the movement of the cell body for about 1.4 minutes (the average *τ* is at 1.38 ± 0.14 minutes based on 4 different samples). Also, the amplitudes of the cross-correlation function do not show the significant decay as a function of time, implying the order relationship of the two waves stands not only for a single event but rather maintains with a repeating cycle. From this result, it suggests that the cell body movement seems to correlate with dorsal actin wave dynamics, or at least the migration of the cells during their “run” stage shows correlation with the dorsal waves.

**Fig 4:**
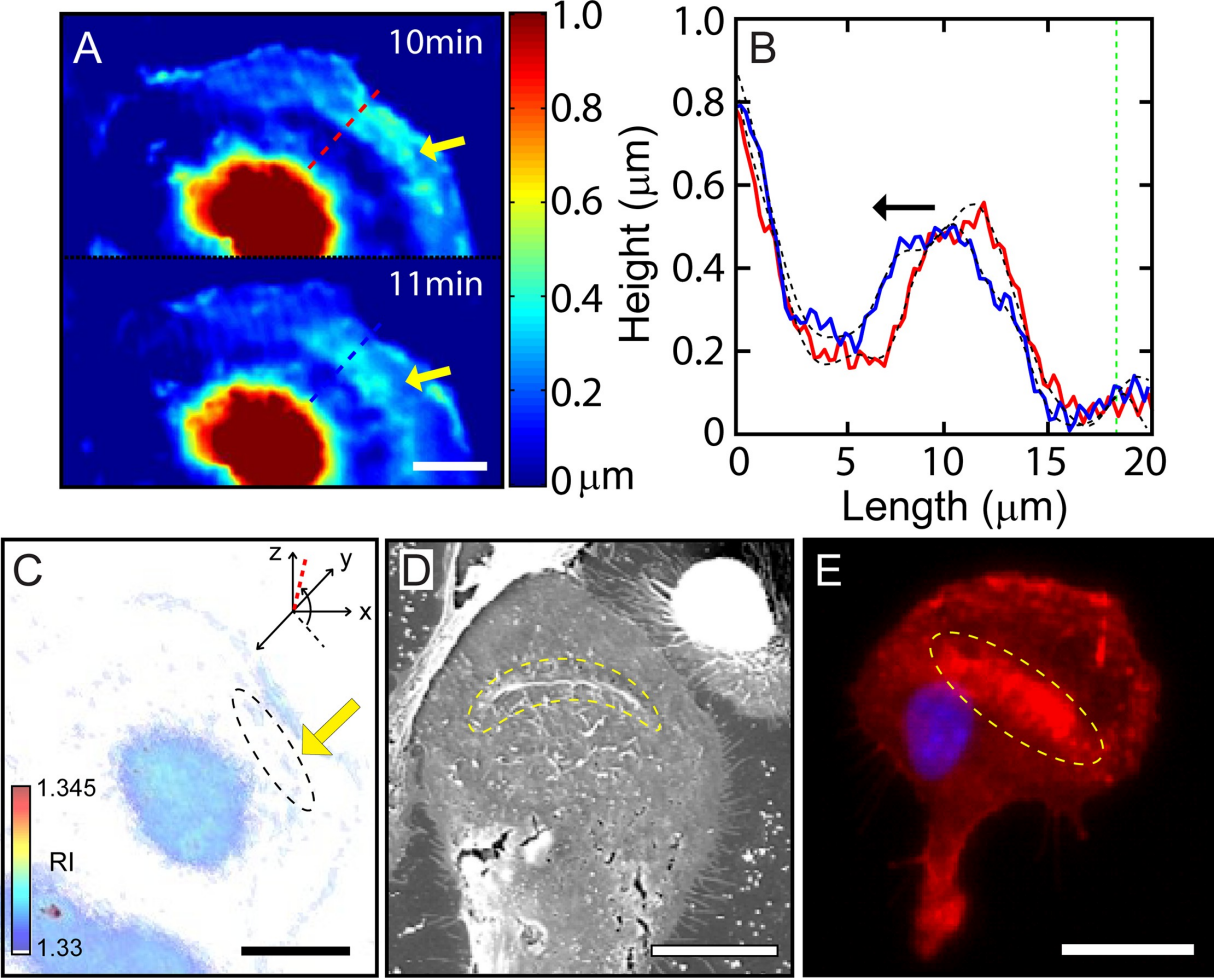
Quantification of periodic dorsal-fin shaped waves. A) Phase image of SAM showing dorsal-fin shaped ruffle in the lamella indicated by yellow arrows. B) 1-D plot of cell surface from bottom showing along the red and blue dotted lines drawn in (A). Black-dotted lines show smoothed values using a low-pass filter with 6 points and green dotted line represents a cell boundary. Black arrow denotes the direction of dorsal wave propagation. C) An isosurface image of 3-D rendered of RI tomogram (see Visualization 5). Black dotted area and yellow arrow indicate the dorsal fin-shaped ruffle in the lamellipodium region (Tomogram displays the viewpoint with azimuth and elevation to -137 º and 84 º, respectively). D) SEM image shows dorsal wave formed in the middle of lamella in the fixed microglia indicated yellow dotted area. E) FM image representing stained by DAPI (blue) and Phalloidin (red) for nucleus and F-actin, respectively. The yellow dotted area indicates the region of concentrated F-actin. Scale bars in A) and C) are 10 μm. They are 20 μm in D) and E).

As done for peripheral ruffle waves in previous section, we use several different imaging modalities to quantify these dorsal waves supported by crawling MG cells. First, we use a QPI technique in conjunction with SAM to obtain heat maps of the height of the dorsal surface shown in Fig 4A. Clearly, these two heat maps suggest the formation of a dorsal wave (indicated by yellow arrows) near the leading front and its propagation toward the core region. Height profiles of the dorsal membrane along the direction of its propagation are plotted in Fig 4B at two different time points t = 10 and 11 minutes, with red and blue lines, respectively. Two black dotted lines show smoothed curves using a low-pass filter with 6 points and the green dotted line represents the cell boundary. The propagation of the dorsal wave towards the cell core is rather clear.

For this cell, the dorsal waves are estimated the propagation speed of 2.5 ± 0.7 μm/min (based on 5 cases). And the height, width (FWHM) and length of dorsal waves are also obtained as 0.48 ± 0.05 μm, 7.37 ± 0.47 μm, and 46.78 ± 8.77 μm (based on 5 cases), respectively. Thus, dorsal waves are far broader than peripheral ruffle waves. A 3-D rendering of RI tomogram shown in Fig 4C, obtained by ODT using a tomographic algorithm (see Visualization 5). It also confirms the existence of a dorsal wave (encircled by a black dashed line and marked by a yellow arrow). The existence of dorsal waves is also evident in the SEM image shown in Fig 4D.

In addition, we stained a sample of MG cell to identify F-actin distribution in association with dorsal waves. As seen in the peripheral ruffle waves at the leading edge, dorsal waves are also rich in F-actin (red color) as shown in Fig 4E. It is known that actin-rich region within lamellipodia can arise as dissociated actin monomers are recruited and pulled toward cell body by myosin-based contractile force [34, 35].

### Synchronized peripheral ruffles waves existing at the leading edge in turning-inhibited cells

**Fig 5:**
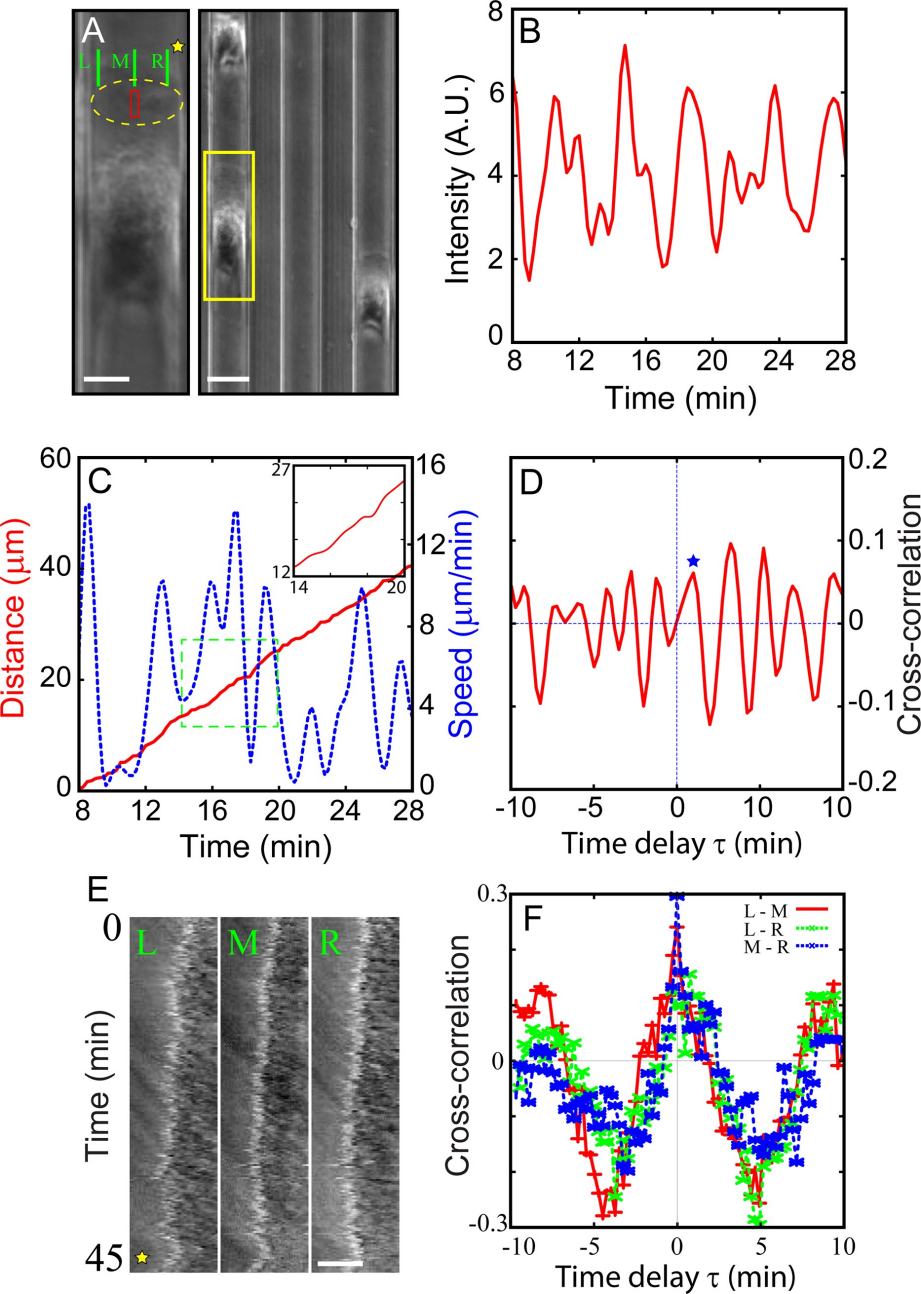
Periodic dorsal actin waves supported by MG cells crawling along quasi-1D tracks made by PDMS. Physically, turns are inhibited. (A) A snapshot image showing three MG cells moving (up) along the tracks (or grooves) that are micro-fabricated (right). (left) A blown-up image of the yellow-boxed area. The area bounded by yellow dashed line includes a dorsal actin wave (see Visualization 6). B) Average intensity of the red rectangular area in A) vs. time, showing a noisy, yet periodic nature of dorsal actin waves. C) The distance travelled by cell (core) (red line) and the moving speed of the cell (blue dotted line). The inset is simply a close-up view of the green boxed area. D) The cross-correlation function between the travelling intensity fluctuation of a dorsal actin wave and the moving speed of the cell body. The star symbol marks the first significant peak position. E) Kymograph are taken alongside (left, middle, and right) the green lines in (A) and the yellow star marks the space outside the cell. F) The cross-correlation functions were calculated using left-middle (red line), left-right (green dotted line), and middle-right (blue dotted line), respectively. Scale bars in left and right of A) are 10 μm, 20 μm and E) 5 μm, respectively.

According to the cross-correlation analysis discussed in the previous section, it is rather clear that the run stage of MG cell crawling is correlated with periodic dorsal actin waves. To understand more clearly about the relationship between cell body movements and “dorsal waves”, we try to inhibit the turning events. Unfortunately, at this moment we do not know how to manipulate only the position of “peripheral ruffles” locally around the cell perimeter without disrupting other cytoskeletal components that are involved in the cell motility, specifically in our case “runs” and “turns” of cells (in Figs 2F and 4E). The usage of any drugs or chemicals known to either block or stimulate the actin dynamics would not have a specific control for these “runs” and “turns” of cells. Thus, we decide to take the drug/chemical nonintrusive route where the micro-patterned 1-D track (defined by a 3-D PDMS groove: ridge height 4 μm, track width 20 μm; see Fig 5A) is used to make turning-inhibited cells with physical confinement. Fortunately, fully physically confined cells by the track width indeed go straight without any turns (see Visualization 6). We demonstrate the peripheral ruffles do exist in the crawling direction of the cell even when the cell can only move to one direction. Here, ‘no turn’ simply means ‘no biased localization of peripheral ruffles’, not the peripheral ruffles completely disappearing from the cell.

With the turning events being prevented, it is impossible to observe the active zone of peripheral ruffles showing any bias (either to the right or left side) of the cell. But the peripheral ruffles still exist at the leading front of the crawling cell and seem to be spatial-temporally synchronized along the active zone of peripheral ruffles. In other words, there are no travelling back and forth (as biased) of peripheral ruffles along the leading front. In Fig 5E, the kymographs are taken from left, middle, and right sides along the leading edge indicated by green lines (as L, M, and R in Fig 5A, and the yellow star marks in Fig 5A and 5E represent the same location). To calculate the cross-correlation functions of ruffling dynamics at the leading edge, we trace the edge position in kymograph using the binarized and skeletonized image analysis of ImageJ. The cross-correlation functions of peripheral ruffles at the leading edge are calculated: left-middle (red line), left-right (green dotted line), and middle-right (blue dotted line) in Fig 5F. The primary peak shows up at 0 delay time, indicating when the cells crawling forward, peripheral ruffles are formed concurrently at the leading edge positions (L, M, and R), expressed as the term “phase-coherent”.

Interestingly, there are two waves with similar period of about 2 minutes in the 1-D track: the dorsal waves (1.89 ± 0.52 min) and the periodic cell movement (1.80 ± 0.72 min). And, we quantify the order relationship of these two oscillatory series (shown in Fig 5B and 5C). In Fig 5D, the correlation function has a primary peak (marked by a blue star) at around *τ* = 1.2 minutes (1.19 ± 0.59 min, based on 4 cases), meaning the dorsal wave propagation (at the red-boxed area in Fig 5A) precede the movement of the cell body for about 1.2 minutes. Also, the amplitudes of the cross-correlation function do not show the significant decay as a function of time, implying the order relationship of the two waves is not considered as a single event but rather maintains with a repeating cycle. With respect to the time delay *τ*, there is no significant difference between the results from 1-D (turning-inhibited, *τ* = 1.2) and 2-D (with turning, *τ* = 1.3). From these results (in 1-D and 2-D), the cell body movement seems to correlate with dorsal actin wave dynamics, or at least correlated with the migration of the cells during their “run” stage.

## Discussion

Cell crawling is a complex process that requires collective movement and coordination of various cytoskeletal elements including actin and myosin. While a lot is known about the molecular pathways that regulate actin-based cell motility [36], the mesoscopic collective behavior of the cytoskeletal elements is much less studied. We decided that the interesting zigzag motile behavior of crawling microglia could be a good example to be understood from the view point of collective actomyosin dynamics. Subsequently, we used several non-invasive quantitative phase imaging modalities to visualize 3-D actin waves inside crawling cells in action. We found that there are two different types of actin waves associated with different actions. One type was peripheral ruffle waves determining the direction of new turn while the other type was dorsal actin waves guiding (or pulling) the cell body forward during the run stage.

These peripheral ruffle waves of MG cells were in many ways very similar to the “lateral membrane waves” analyzed earlier by Dobereiner *et*. *al*. [37] who quantified lateral membrane waves supported by a number of different cells. They found that there could be many different types of spatiotemporal evolution of them, including a periodic oscillation. Giannone *et*. *al*. have reported that the periodicity of the lateral waves was closely associated with the period of protrusion and retraction cycle of the leading front [23]. Both studies foresaw the significance of lateral waves in cell motility. However, they did not discuss the specific role of these waves. In this report, we demonstrated that peripheral ruffle waves that are essentially lateral waves could be considered as a precursory activity setting the direction of new turn for crawling MG cells.

Peripheral ruffles were actin-based, very narrow, vertical protrusions that constantly form and collapse to fold back on themselves. They usually only formed at the leading front. Besides these peripheral ruffles, there were other known types of actin-based waves, for example, circular dorsal ruffles (or CDRs in short) [38, 39]. CDRs were fronts of actin polymerization moving toward the cell core over the dorsal membrane surface, more or less in a form of circle. They had a very significant biological role in endocytosis. These dorsal actin waves that we report in this work are quite similar to CDRs since they also protruded over the dorsal surface and moved toward the core. Hence, we claimed these dorsal waves to be responsible for the run stage in which the role of dorsal actin waves was considered as a pulling (or coordinating) force (or signal) for persistently directed crawling of MG cell as the periodic dorsal actin wave dynamics showed a correlation with the pulsatile movement of the cell body. Also, the quasi-1D track experiment discussed in Fig 5 demonstrated “no swinging activity” of peripheral ruffles, implying no turning. Thus, by inhibiting any turns with a physical confinement, the ruffle activities were still present at the leading edge with phase coherence. Unfortunately, at this moment we do not know how to manipulate only the position of “peripheral ruffles” locally around the cell perimeter without disrupting other cytoskeletal components that are involved in the cell motility.

Although both ruffles at the leading front and dorsal actin waves exhibited a quite regular oscillatory behavior, their periods were at least one order of magnitude different. What sets these two-time scales different was currently unclear. As discussed by Weiner *et*. *al*. [40], it was likely that there should be an underlying regulator of actin waves that is expressed differently for different locations within the cell body. Giannone *et*. *al*. have also found that the period could be changed by extrinsic factors such as stiffness of the substrate [23].

In conclusion, using several advanced imaging techniques such as SAM, ODT of high-sensitivity QPI, PCM, SEM, and FM, we could have observed two different types of collective actin wave dynamics associated with cell crawling. One type was peripheral ruffle waves while the other type was dorsal actin waves. They were different in sizes (height: ~ 0.4 μm and ~ 0.5 μm, width: ~ 0.4 μm and ~ 7.4 μm, and length: ~ 9.0 μm and 46.8 μm) and temporal dynamics (period: ~6 s vs. ~2 min) as shown in Table 1. Most importantly, we identified different roles that they undertook for the crawling of MG cells, namely, turning and forward movement. The idea that cell motility results from coordinated activity of multiple waves was not new. However, it became increasingly more important as discussed in Refs. 40 and 41 [40, 41]. To pursue this idea in practice, we needed non-invasive, highly accurate, precise, and fast 3-D imaging tools and techniques. For that matter, innovative 3-D phase optics that we employed in this work would be of great use in the future alongside other imaging schemes. Finally, what is not known at the moment is the molecular-level coordination between the two different actin wave activities working at two quite different time and space scales: In other words, how ‘turns’ and ‘runs’ join smoothly.

**Table 1:** The properties of the peripheral ruffle waves and dorsal wave.

	Period	Speed (μm/min)	Height (μm)	Width (μm)	Length (μm)
Peripheral ruffle wave	6.45 ± 0.44 s	N/A	0.40 ± 0.13	0.39 ± 0.09	9.04 ± 2.51
		(no propagation)			
Dorsal ruffle wave	1.91 ± 0.87 m	2.5 ± 0.7	0.48 ± 0.05	7.37 ± 0.47	46.78 ± 8.77

## Supporting information

S1 File Visualization 1. A typical zigzag trajectory with sequential events of turn-and-runs obtained by PCM.
(AVI)

S2 File Visualization 2. Phase image of peripheral ruffles in living microglia obtained by a QPI.
(AVI)

S3 File Visualization 3. Peripheral ruffles in living microglia with isosurface image of 3-D RI tomogram.
(AVI)

S4 File Visualization 4. Periodic dorsal waves in forward migrating cell obtained by PCM.
(AVI)

S5 File Visualization 5. Periodic dorsal waves in microglia with isosurface image of 3-D RI tomogram.
(AVI)

S6 File Visualization 6. Dorsal wave in the absence of any turns obtained by PCM.
(AVI)

S1 Data The raw data of the figures. The raw data of all figures are included in this file.
(XLSX)

## References

[1] Theriot JA, Mitchison TJ. Actin microfilament dynamics in locomoting cells. Nature. 1991;352(6331):126 10.1038/352126a0 2067574

[2] Mullins RD, Heuser JA, Pollard TD. The interaction of Arp2/3 complex with actin: nucleation, high affinity pointed end capping, and formation of branching networks of filaments. Proceedings of the National Academy of Sciences. 1998;95(11):6181–6. 10.1073/pnas.95.11.6181 PMC27619 9600938

[3] Ghosh M, Song X, Mouneimne G, Sidani M, Lawrence DS, Condeelis JS. Cofilin promotes actin polymerization and defines the direction of cell motility. Science. 2004;304(5671):743–6. 10.1126/science.1094561 15118165

[4] Ponti A, Machacek M, Gupton S, Waterman-Storer C, Danuser G. Two distinct actin networks drive the protrusion of migrating cells. Science. 2004;305(5691):1782–6. 10.1126/science.1100533 15375270

[5] Yang TD, Park JS, Choi Y, Choi W, Ko TW, Lee KJ. Zigzag turning preference of freely crawling cells. PLoS One. 2011;6(6):e20255 10.1371/journal.pone.0020255 21687729 PMC3110194

[6] Barnhart EL, Allen GM, Jülicher F, Theriot JA. Bipedal Locomotion in Crawling Cells. Biophysical Journal. 2010;98(6):933–42. 10.1016/j.bpj.2009.10.058 20303850 PMC2849068

[7] Li L, Nørrelykke SF, Cox EC. Persistent Cell Motion in the Absence of External Signals: A Search Strategy for Eukaryotic Cells. PLoS ONE. 2008;3(5):e2093 10.1371/journal.pone.0002093 18461173 PMC2358978

[8] Lauffenburger DA, Horwitz AF. Cell Migration: A Physically Integrated Molecular Process. Cell. 1996;84(3):359–69. 10.1016/s0092-8674(00)81280-5 8608589

[9] Lee J, Jacobson K. The composition and dynamics of cell-substratum adhesions in locomoting fish keratocytes.12. 10.1242/jcs.110.22.2833 9427291

[10] Shutova M, Yang C, Vasiliev JM, Svitkina T. Functions of Nonmuscle Myosin II in Assembly of the Cellular Contractile System. PLoS ONE. 2012;7(7):e40814 10.1371/journal.pone.0040814 22808267 PMC3396643

[11] Sastry SK, Burridge K. Focal Adhesions: A Nexus for Intracellular Signaling and Cytoskeletal Dynamics. Experimental Cell Research. 2000;261(1):25–36. 10.1006/excr.2000.5043 11082272

[12] DeMali KA. Coupling membrane protrusion and cell adhesion. Journal of Cell Science. 2003;116(12):2389–97. 10.1242/jcs.00605 12766185

[13] Krause M, Gautreau A. Steering cell migration: lamellipodium dynamics and the regulation of directional persistence. Nature reviews Molecular cell biology. 2014;15(9):577 10.1038/nrm3861 25145849

[14] Ladwein M, Rottner K. On the Rho’d: the regulation of membrane protrusions by Rho-GTPases. FEBS letters. 2008;582(14):2066–74. 10.1016/j.febslet.2008.04.033 18442478

[15] Bernitt E, Koh CG, Gov N, Döbereiner H-G. Dynamics of Actin Waves on Patterned Substrates: A Quantitative Analysis of Circular Dorsal Ruffles. PLOS ONE. 2015;10(1):e0115857 10.1371/journal.pone.0115857 25574668 PMC4289068

[16] Petrie RJ, Doyle AD, Yamada KM. Random versus directionally persistent cell migration. Nature reviews Molecular cell biology. 2009;10(8):538 10.1038/nrm2729 19603038 PMC2752299

[17] Selmeczi D, Mosler S, Hagedorn PH, Larsen NB, Flyvbjerg H. Cell Motility as Persistent Random Motion: Theories from Experiments. Biophysical Journal. 2005;89(2):912–31. 10.1529/biophysj.105.061150 15951372 PMC1366641

[18] Choi Y, Kim M, Yoon C, Yang TD, Lee KJ, Choi W. Synthetic aperture microscopy for high resolution imaging through a turbid medium. Optics Letters. 2011;36(21):4263 10.1364/OL.36.004263 22048385

[19] Kim M, Choi Y, Fang-Yen C, Sung Y, Dasari RR, Feld MS, et al High-speed synthetic aperture microscopy for live cell imaging. Optics Letters. 2011;36(2):148 10.1364/OL.36.000148 21263482 PMC3068016

[20] Choi W, Fang-Yen C, Badizadegan K, Oh S, Lue N, Dasari RR, et al Tomographic phase microscopy. Nature Methods. 2007;4(9):717–9. 10.1038/nmeth1078 17694065

[21] Sung Y, Choi W, Fang-Yen C, Badizadegan K, Dasari RR, Feld MS. Optical diffraction tomography for high resolution live cell imaging. 2009:12. 10.1364/oe.17.000266 PMC2832333 19129896

[22] Bosgraaf L, Van Haastert PJM. The Ordered Extension of Pseudopodia by Amoeboid Cells in the Absence of External Cues. PLoS ONE. 2009;4(4):e5253 10.1371/journal.pone.0005253 19384419 PMC2668753

[23] Giannone G, Dubin-Thaler BJ, Döbereiner H-G, Kieffer N, Bresnick AR, Sheetz MP. Periodic Lamellipodial Contractions Correlate with Rearward Actin Waves. Cell. 2004;116(3):431–43. 10.1016/s0092-8674(04)00058-3 15016377

[24] Heinisch JJ, Lipke PN, Beaussart A, El Kirat Chatel S, Dupres V, Alsteens D, et al Atomic force microscopy—looking at mechanosensors on the cell surface. Journal of Cell Science. 2012;125(18):4189–95. 10.1242/jcs.106005 23077172 PMC3516434

[25] Rilla K, Koistinen A. Correlative Light and Electron Microscopy Reveals the HAS3-Induced Dorsal Plasma Membrane Ruffles. Int J Cell Biol. 2015;2015:769163 10.1155/2015/769163 26448759 PMC4581547

[26] Kim G, Lee M, Youn S, Lee E, Kwon D, Shin J, et al Measurements of three-dimensional refractive index tomography and membrane deformability of live erythrocytes from Pelophylax nigromaculatus. Scientific Reports. 2018;8(1). 10.1038/s41598-018-25886-8 29907826 PMC6003953

[27] Lee S, Park H, Kim K, Sohn Y, Jang S, Park Y. Refractive index tomograms and dynamic membrane fluctuations of red blood cells from patients with diabetes mellitus. Scientific Reports. 2017;7(1). 10.1038/s41598-017-00035-9 28432323 PMC5430658

[28] Park Y, Best CA, Auth T, Gov NS, Safran SA, Popescu G, et al Metabolic remodeling of the human red blood cell membrane. Proceedings of the National Academy of Sciences. 2010;107(4):1289–94. 10.1073/pnas.0910785107 20080583 PMC2802590

[29] Jourdain P, Pavillon N, Moratal C, Boss D, Rappaz B, Depeursinge C, et al Determination of Transmembrane Water Fluxes in Neurons Elicited by Glutamate Ionotropic Receptors and by the Cotransporters KCC2 and NKCC1: A Digital Holographic Microscopy Study. Journal of Neuroscience. 2011;31(33):11846–54. 10.1523/JNEUROSCI.0286-11.2011 21849545 PMC6623187

[30] Wang Z, Millet L, Mir M, Ding H, Unarunotai S, Rogers J, et al Spatial light interference microscopy (SLIM). 2011:11. 10.1364/OE.19.001016 PMC3482902 21263640

[31] Mir M, Wang Z, Shen Z, Bednarz M, Bashir R, Golding I, et al Optical measurement of cycle-dependent cell growth. Proceedings of the National Academy of Sciences. 2011;108(32):13124–9. 10.1073/pnas.1100506108 21788503 PMC3156192

[32] Aknoun S, Savatier J, Bon P, Galland F, Abdeladim L, Wattellier B, et al Living cell dry mass measurement using quantitative phase imaging with quadriwave lateral shearing interferometry: an accuracy and sensitivity discussion. Journal of Biomedical Optics. 2015;20(12):126009 10.1117/1.JBO.20.12.126009 26720876

[33] Wang Z. Tissue refractive index as marker of disease. Journal of Biomedical Optics. 2011;16(11):116017 10.1117/1.3656732 22112122 PMC3223513

[34] Machacek M, Danuser G. Morphodynamic profiling of protrusion phenotypes. Biophys J. 2006;90(4):1439–52. 10.1529/biophysj.105.070383 16326902 PMC1367294

[35] Totsukawa G, Wu Y, Sasaki Y, Hartshorne DJ, Yamakita Y, Yamashiro S, et al Distinct roles of MLCK and ROCK in the regulation of membrane protrusions and focal adhesion dynamics during cell migration of fibroblasts. J Cell Biol. 2004;164(3):427–39. 10.1083/jcb.200306172 14757754 PMC2172229

[36] Pollard TD, Borisy GG. Cellular motility driven by assembly and disassembly of actin filaments. Cell. 2003;112(4):453–65. 10.1016/s0092-8674(03)00120-x PubMed PMID: WOS:000181252600005. 12600310

[37] Döbereiner H-G, Dubin-Thaler BJ, Hofman JM, Xenias HS, Sims TN, Giannone G, et al Lateral membrane waves constitute a universal dynamic pattern of motile cells. Physical review letters. 2006;97(3):038102 10.1103/PhysRevLett.97.038102 16907546

[38] Bernitt E, Döbereiner H-G. Spatiotemporal Patterns of Noise-Driven Confined Actin Waves in Living Cells. Physical review letters. 2017;118(4):048102 10.1103/PhysRevLett.118.048102 28186815

[39] Hoon J-L, Wong W-K, Koh C-G. Functions and regulation of circular dorsal ruffles. Molecular and cellular biology. 2012:MCB. 00551–12. 10.1128/MCB.00551-12 PMC3486146 22927640

[40] Weiner OD, Marganski WA, Wu LF, Altschuler SJ, Kirschner MW. An actin-based wave generator organizes cell motility. PLoS biology. 2007;5(9):e221 10.1371/journal.pbio.0050221 17696648 PMC1945041

[41] Dreher A, Aranson IS, Kruse K. Spiral actin-polymerization waves can generate amoeboidal cell crawling. New Journal of Physics. 2014;16(5):055007.

