# Correction: Two distinct actin waves correlated with turns-and-runs of crawling microglia

**DOI:** 10.1371/journal.pone.0222692

**Published:** 2019-09-12

**Authors:** Taeseok Daniel Yang, Kwanjun Park, Jin-Sung Park, Jang-Hoon Lee, Eunpyo Choi, Jonghwan Lee, Wonshik Choi, Youngwoon Choi, Kyoung J. Lee

The following information is missing from the Funding statement: This work was supported in part by the National Research Foundation of Korea Grant 2016R1D1A1B03930591 to KJL.
