# Correction: Correction: Two distinct actin waves correlated with turns-and-runs of crawling microglia

**DOI:** 10.1371/journal.pone.0229814

**Published:** 2020-02-26

**Authors:** Taeseok Daniel Yang, Kwanjun Park, Jin-Sung Park, Jang-Hoon Lee, Eunpyo Choi, Jonghwan Lee, Wonshik Choi, Youngwoon Choi, Kyoung J. Lee

There is an error in the Correction published on September 12, 2019. The correct grant number is 2019R1A2C2005989. The correct Funding statement is: This work was supported in part by the National Research Foundation of Korea Grant 2019R1A2C2005989 to KJL.
